# Immune checkpoint inhibitor-induced multi-organ immune damage complicated by severe pneumonia: case report

**DOI:** 10.3389/fimmu.2026.2003924

**Published:** 2026-10-21

**Authors:** Tingrui Zhao, Rongzhang Chen, Yuhua Ma, Gen Li, Xia Gu, Yukai Chen, He Sun

**Affiliations:** 1 Department of Clinical Pharmacy, The Third Hospital of Mianyang, Sichuan Mental Health Center, Mianyang, China; 2 Department of Respiratory and Critical Care Medicine, Shanghai East Hospital, Tongji University School of Medicine, Shanghai, China; 3 Department of General Practice, Shanghai East Hospital, Tongji University School of Medicine, Shanghai, China; 4 Department of Clinical Laboratory, Shanghai East Hospital, Tongji University School of Medicine, Shanghai, China

**Keywords:** hypophysitis, immune checkpoint inhibitors, immune-related adverse events, multi-drug resistant bacteria, serplulimab

## Abstract

Immune checkpoint inhibitor (ICI) have substantially extended survival in patients with extensive-stage small-cell lung cancer (ES-SCLC), yet they also carry the risk of life-threatening immune-related adverse events (irAEs). Clinical experience in managing multi-organ irAEs (MO-irAEs) complicated by respiratory failure and subsequent severe pneumonia remains exceedingly scarce. Here we report a 71-year-old patient with ES-SCLC (stage IVb, with brain and splenic metastases) who, after four cycles of serplulimab plus chemotherapy, sequentially developed myocarditis, myositis, and hepatic injury. Following premature tapering of glucocorticoid therapy, the patient experienced a recurrence of ICI-related toxicity, manifesting as hypophysitis, primary hypothyroidism, and adrenal insufficiency-alongside respiratory failure-and later acquired multidrug-resistant infection during the treatment course. Through individualized moderate-dose glucocorticoids, comprehensive hormone replacement, and pathogen-directed antimicrobial therapy with eravacycline and ceftazidime-avibactam guided by metagenomic next-generation sequencing (mNGS), the infection was ultimately brought under control and multi-organ dysfunction reversed. This case underscores several key points: (1) serplulimab can precipitate multi-endocrine gland insufficiency, and severe hypothyroidism may be one of the factors contributing to the respiratory failure, mandating a high index of clinical suspicion; (2) glucocorticoid regimens should adhere to adequate initial dosing, sufficient duration, and slow tapering-abrupt discontinuation risks triggering recurrence of ICI-related toxicity; and (3) rapid, precise lower-respiratory-tract pathogen identification coupled with targeted antimicrobial therapy offers particular value in managing severe pneumonia caused by drug-resistant organisms.

## Introduction

Small cell lung cancer (SCLC) accounts for 13–15% of lung cancers and is the most aggressive subtype, with rapid proliferation, strong invasiveness, and early distant spread. At initial diagnosis, 60–70% of patients have extensive-stage disease, and roughly two-thirds have distant metastases; those with brain or liver involvement have a particularly poor prognosis (1). Although the tumor responds well to first-line chemoradiotherapy, most patients relapse and become resistant within a short time, yielding a 5-year overall survival below 7% (2).

ICI, such as serplulimab, restore T-cell-mediated antitumor immune responses and have become an important treatment modality for ES-SCLC (3, 4). With its increasingly widespread clinical use, reports of associated irAEs have also been accumulating.

However, ICI can also break immune tolerance while boosting antitumor immunity, resulting in irAEs involving diverse organs such as the skin, gastrointestinal tract, lung, heart, liver, and endocrine glands. When several organ systems are affected at the same time, particularly in the presence of respiratory failure, the overlapping and non-specific clinical features pose major challenges to both diagnosis and management (5). Here we report a case of successful rescue in a patient who developed MO-irAEs-including myocarditis, myositis, hepatic injury, and multi-endocrine gland damage-after serplulimab treatment, followed by a multidrug-resistant infection, with the aim of providing a reference for the clinical management of similar critical cases.

## Case presentation

The patient was a 71-year-old man weighing 65 kg, with a history of smoking and no history of diabetes, thyroid disease, or autoimmune disease. In August 2024, he presented with cough and dyspnea. Bronchoscopic biopsy pathology confirmed right lung small cell lung cancer, clinical stage IVb (extensive stage), with splenic and brain metastases, and an ECOG performance status score of 1. From September to December 2024, he completed four cycles of chemotherapy combined with immunotherapy at another institution (regimen: etoposide 0.194 g on days 1-3, lobaplatin 40 mg on day 1, and serplulimab 300 mg on day 1, administered every 3 weeks). During treatment, he experienced fatigue, which was not specifically managed. In January 2025, antitumor therapy was suspended due to liver and kidney injury, and the patient was discharged after hepatoprotective treatment.

On January 22, the patient was admitted to the VIP ward because of progressive generalized weakness, poor appetite, and fecal and urinary incontinence. Laboratory findings on admission revealed the following: alanine aminotransferase(ALT) 124 IU/L, aspartate aminotransferase(AST) 239.9 IU/L, lactatedehydrogenase(LDH) 667.2 U/L, serum creatinine 115 μmol/L, creatine kinase (CK) 1727 U/L, myoglobin(Mb) 576.8 ng/mL, high-sensitivity cardiac troponin T (hs-TnT) 0.102 ng/mL, creatine kinase-MB(CK-MB) 25.25 ng/mL. Chest CT (**Figure 1A**) demonstrated complete remission (CR) of the lung cancer, along with emphysema, scattered multifocal inflammation in both lungs, and bilateral pleural effusion. He was diagnosed with immune checkpoint inhibitor-related multiorgan injury (myocarditis, myositis, liver injury, kidney injury), graded 3–4 according to Common Terminology Criteria for Adverse Events(CTCAE) criteria, and treated with methylprednisolone 80 mg/d, with gradual tapering.

**Figure 1:**
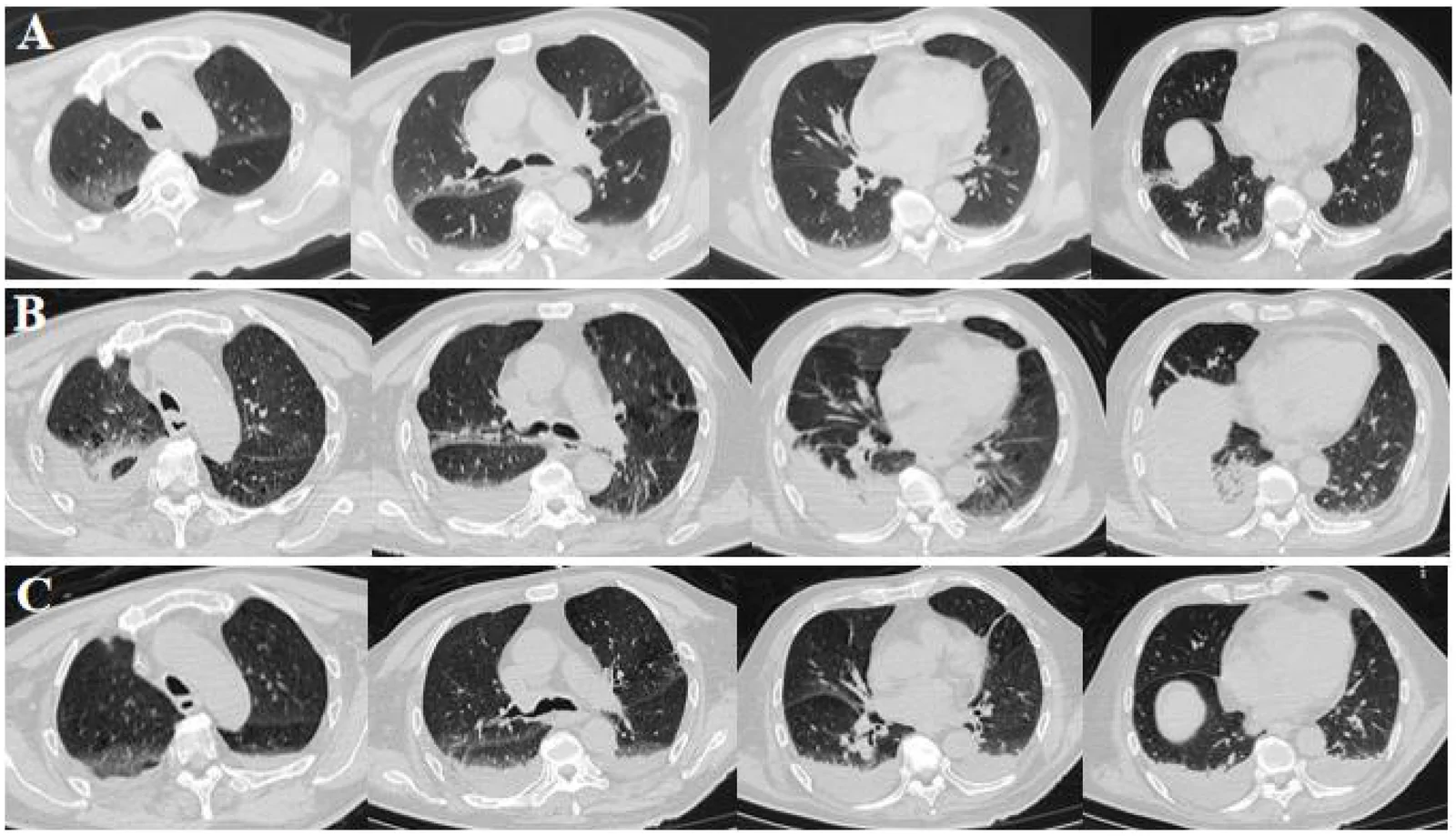
**(A)** Chest CT on January 22, 2025, one month prior to admission, revealed a complete remission (CR) of the lung cancer. There were emphysemas, scattered inflammatory infiltrations, and bilateral pleural effusion. **(B)** Chest CT on February 26, 2025, revealed that the cancer remained CR. There was pleural effusion on the right, accompanied by right lower lobe atelectasis and consolidation. **(C)** Follow-up chest CT on March 18, 2025, showing some absorption of the pulmonary lesions compared with the previous CT, with only a small amount of bilateral pleural effusion remaining.

On February 26, 2025, the patient was admitted again because of “worsening generalized fatigue, lethargy, and bilateral lower limb edema”. Physical examination revealed confusion, lethargy, macroglossia with posterior tongue base collapse, hyperpigmented and indurated skin, and pitting edema of both lower extremities. During this hospitalization, he developed respiratory failure (pH 7.27, PaO_2_ 62.2 mmHg, PaCO_2_ 86.5 mmHg) and was transferred to the respiratory intensive care unit(RICU). The patient’s family declined endotracheal intubation; consequently, a nasopharyngeal airway was placed, and high-flow nasal oxygen was administered for respiratory support.

Admission laboratory results were categorized by organ system as follows:

(1) Inflammatory and infectious markers: white blood cell count(WBC) 5.63×10^9^/L, neutrophil percentage(NEUT%) 84.5%, C-reactive protein(CRP) 78.78 mg/L, serum amyloid A(SAA) 145.91 mg/L, procalcitonin(PCT) 0.12 ng/mL, interleukin-6(IL-6) 32.86 pg/mL, CD4^+^ T-cell count 240 cells/μL, CD8^+^ T-cell count 107.77 cells/μL, and serum G/GM tests were negative.

(2) Cardiac injury markers: CK 438 U/L, hs-TnT 0.29 ng/mL, CK-MB 19.22 ng/mL, Mb 133.6 ng/mL, and BNP 239.4 ng/L.

(3) Hepatic, renal, and electrolyte parameters: ALT 50 U/L, AST 49.5 U/L, alkaline phosphatase(ALP) 73.5 U/L, Scr 88.3 μmol/L, serum K^+^ 3.87 mmol/L, serum Na^+^ 124.58 mmol/L.

(4) Endocrine hormone panel (**Table 1**, **Supplementary Tables S1, S2**):

**Table 1:** Serial laboratory endocrine and myocardial biomarker test results of the patient.

Indicator	COR (μg/dL)	ACTH (pg/mL)	GH (pg/mL)	FSH (mIU/mL)	PRL (ng/mL)	LH (mIU/mL)	T (ng/mL)	T4 (μg/dL)	TSH (μIU/mL)	CK (U/L)	hs-TnT (ng/mL)	CK-MB (ng/mL)
Normal range	7-9AM: 4.26-24.85	7-9AM:7.2-63.4	0.03-2.47	1.5-12.4	4.04-15.2	1.7-8.6	1.93-7.4	5.1-14.1	0.27-4.2	50-310	<0.014	<4.87
02/27/25	26.94	5.748	1.27	11.2	1.86	3.36	0.254	<0.42	20.2	192	0.181	7.28
03/05/25	2.956		0.819	12.1	19	1.75	0.598	1	99	98	0.137	3.73
03/11/25	2.086	<1	0.422	11.2	16.3	3.24	0.385	1.4	51.3	40	0.086	1.96
03/27/25	9.485	1.839	0.692	2.32	13.6	<0.3	1.88	6.89	1.1		0.037	0.7

COR, Cortisol; ACTH, Adrenocor ticotropic hormore; GH, Growth Hormone; FSH, Follicle stimulating hormone; PRL, Prolactin; LH, Luteinizing hormone; T, Testosterone; T4, Thyroxine; TSH, Thyrotropin; CK, Creatine Kinase; hs-TnT, High-sensitivity troponin T; CK-MB, Creatine Kinase-MB.

Thyroid profile: triiodothyronine (T3) <0.3 ng/mL, thyroxine (T4) <0.42 μg/dL, free triiodothyronine (FT3) <0.39 pg/mL, free thyroxine (FT4) <0.04 ng/dL, thyroid-stimulating hormone (TSH) 20.2 μIU/mL, thyroglobulin (Tg) <0.04 ng/mL, anti-thyroglobulin antibody (TgAb) 31.6 IU/mL, anti-thyroid peroxidase antibody (TPOAb) 239 IU/mL, and thyrotropin receptor-stimulating antibody (TRAb) 4.49 IU/L.

Adrenal axis: cortisol 26.94 μg/dL and adrenocorticotropic hormone (ACTH) 5.75 pg/mL.

Gonadal and growth hormone axes: growth hormone (GH) 1.27 pg/mL, follicle-stimulating hormone (FSH) 11.2 mIU/mL, prolactin (PRL) 1.86 ng/mL, luteinizing hormone (LH) 3.36 mIU/mL, testosterone 0.25 ng/mL, progesterone 0.28 ng/mL, estradiol 38.9 pg/mL, and insulin-like growth factor-1 (IGF-1) 18.3 ng/mL.

(5) Immunological screening: viral hepatitis screening was negative; complement C3 and C4 were within normal limits; antinuclear antibodies were positive at 1:100 and 1:320; ANCA-related antibodies were all negative; autoimmune myositis antibody panel was negative; and immunoglobulin levels were IgG 9.83 g/L, IgM 0.77 g/L, and IgA 1.53 g/L.

Electromyography revealed myopathic changes. Abdominal CT showed cirrhotic changes with ascites and diffuse thickening of the colonic wall. Cranial CT demonstrated multiple ischemic and encephalomalacic foci in the bilateral basal ganglia. Chest CT (**Figure 1B**) confirmed CR of the lung cancer, with emphysema, scattered exudative changes in both lungs, right-sided pleural effusion, and right lower lobe atelectasis with consolidation. Echocardiography revealed left atrial enlargement, basal septal hypertrophy, and a small pericardial effusion. Electrocardiography showed sinus rhythm, first-degree atrioventricular block, and widened P waves. Bronchoscopy with bronchoalveolar lavage was performed, and lavage fluid was sent for further analysis.

The final diagnoses were immune checkpoint inhibitor-related multi-organ injury (involving myocarditis, myositis, hepatic injury, and renal impairment), clinically suspected hypophysitis, primary thyroiditis, secondary adrenal insufficiency, hypogonadotropic hypogonadism, and type II respiratory failure.

After admission, methylprednisolone 40 mg/d was given to control hypophysitis and the systemic immune reaction. Endocrine replacement therapy was initiated simultaneously: for hypothyroidism, levothyroxine sodium was started at 25 μg/d and gradually titrated to 300 μg/d; for hypogonadism, testosterone undecanoate soft capsules were administered as replacement therapy.

During the course of the disease, the patient underwent respiratory pathogen testing; bronchoalveolar lavage fluid (BALF) mNGS detected carbapenem-resistant Acinetobacter baumannii (CRAB) and carbapenem-resistant Klebsiella pneumoniae (CRKP). Based on these results, eravacycline (1 mg/kg q12h ivgtt) and ceftazidime-avibactam (2.5 g q8h ivgtt) was selected for anti-infective therapy.

After treatment, the patient’s mental status improved markedly, lethargy and fatigue gradually resolved, endocrine-related hormone levels returned to normal over time, and inflammatory markers and myocardial injury markers declined (**Tables 1**, **2**, **Supplementary Tables S1-S4**). On March 18, repeat chest CT showed gradual resolution of pulmonary exudation and disappearance of pneumothorax (**Figure 1C**). The patient was eventually weaned from noninvasive respiratory support and discharged in stable condition. The complete clinical course is summarized in **Figure 2**.

**Table 2:** Serial liver, renal, inflammatory and hematological laboratory parameters of the patient.

Indicator	ALT (U/L)	AST (U/L)	LDH (U/L)	Scr (μmol/L)	K+ (mmol/L)	Na+ (mmol/L)	WBC (×10^9^/L)	NEUT (%)	CRP (mg/L)	SAA (mg/L)	PCT (ng/mL)	IL-6 (pg/mL)
Normal range	<50	17-59	120-246	58-110	3.5-5.1	137-145	3.5-9.5	40-75	0-10	<10	<0.046	0-7
02/26/25	50	49.5	355	88.3	3.87	124.58	5.63	84.5	78.78	145.91	0.116	32.86
03/07/25	43	37	282.7	95.7	4.51	136.95	5.93	78.8	8.61	18.39	0.112	15.61
03/17/25	41	31.1	230.5	78.8	4.4	133.51	4.4	69.7	3.19	<8	0.076	<1.5
03/26/25	38	25	227	89.8	4.21	142.76	7	70.8	80.19	195.75	1.73	63.02
04/02/25	27.9	17.2	211	54.4	4.31	141.25	3.91	69.8	4.44	9.3	0.074	9.88

ALT, Alanine aminotransferase; AST, Aspartate aminotransferase; LDH, Lactate dehydrogenase; Scr, Serum creatinine; K^+^, potassium; Na^+^, sodium; WBC, White blood cell count; NEUT%, Neutrophil percentage; CRP, C-reactive protein; SAA, Serum amyloid A; PCT, Procalcitonin; IL-6, Interleukin-6.

**Figure 2:**
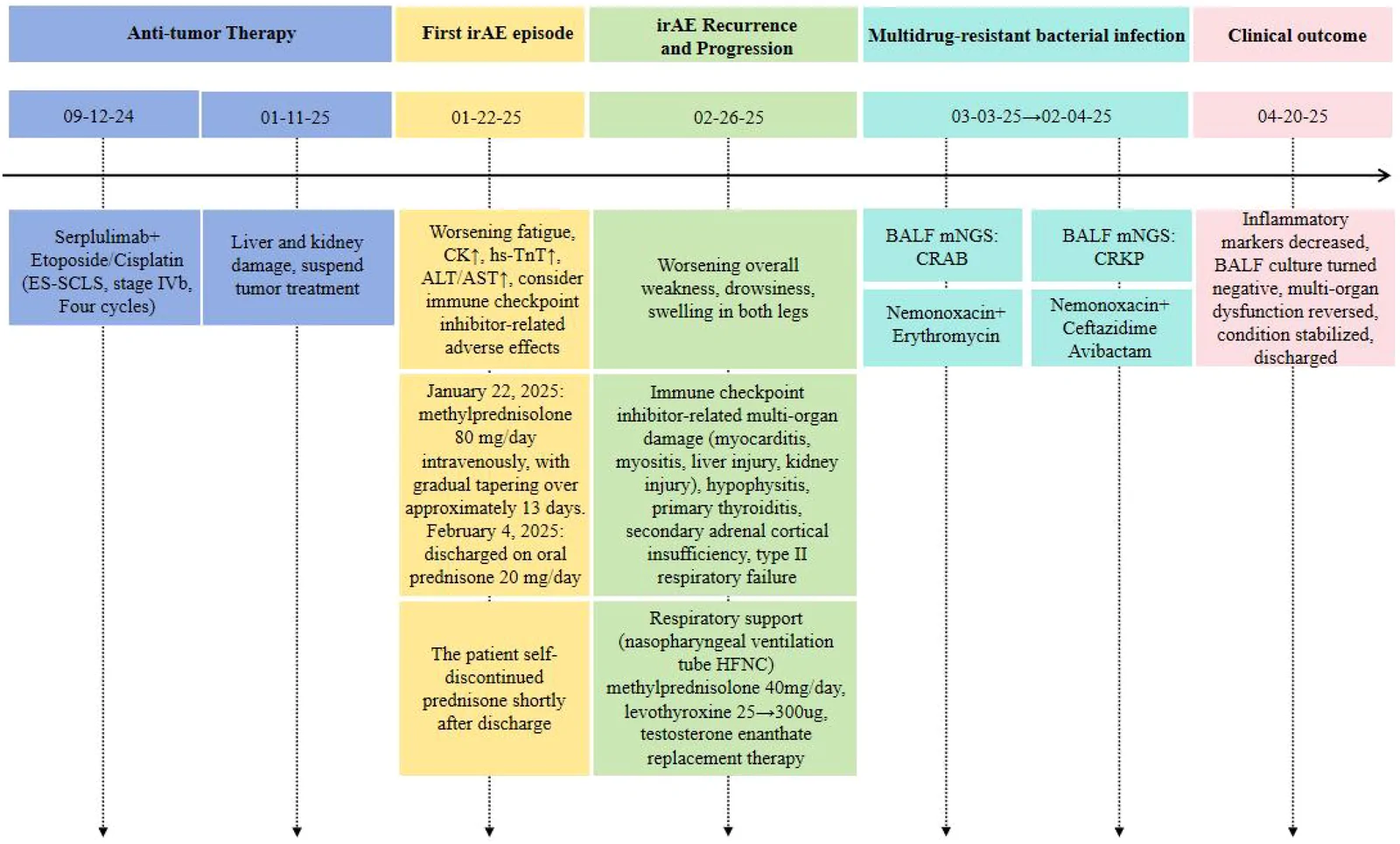
Management timeline of multi-organ irAEs related to serplulimab combined with MDR infection.

## Discussion

IrAEs can affect multiple systems throughout the body (6). In cases involving simultaneous involvement of multiple organs, the severe morbidity rate can reach 26%–41%, substantially higher than that observed with single-organ irAEs (3%–20%) (7). Mortality increases markedly when the heart, skeletal muscle, or endocrine glands are affected (8). Endocrine glands, with their rich blood supply, are frequent target organs for irAEs (9). Persistent fatigue is a common early manifestation (10)and is easily confused with tumor progression, potentially leading to missed diagnosis. This case began with fatigue and progressed to a complex clinical course involving simultaneous involvement of the myocardium, skeletal muscle, liver, kidneys, and multiple endocrine axes. The recognition, treatment, and tapering management have several key points.

The diagnosis of ICI-related hypophysitis remains clinically elusive. While MRI is the preferred imaging modality, pituitary enlargement is often transient (11, 12), with a meta-analysis showing that only 23.7% of affected patients exhibit abnormal enhancement (13). In our patient, MRI was precluded by a metallic dental prosthesis, necessitating a purely biochemical diagnosis. The diagnosis was supported by synchronous impairment of the adrenal (low ACTH/cortisol), gonadal (low testosterone with inappropriately normal LH/FSH), and growth hormone axes, alongside mild hyperprolactinemia suggestive of stalk compression. Crucially, the pattern of multi-axis involvement and the prior history of ICI exposure effectively ruled out metastatic compression (given the basal ganglia location of metastases) and empty sella syndrome, highlighting that in the absence of MRI, a clinical diagnosis based on two or more endocrine axes involvement is both valid and imperative. However, given this patient’s history of glucocorticoid use, exogenous glucocorticoid-induced adrenal insufficiency cannot be entirely ruled out.

In our patient, the combination of elevated TSH, reduced FT4, negative TRAb, and positive TPO-Ab clearly points to primary hypothyroidism rather than central (secondary) hypothyroidism. Most thyroid-related irAEs induced by ICI manifest as overt hypothyroidism, with some patients experiencing a transition from hyperthyroidism to hypothyroidism, which is often permanent. Of note, the patient’s Tg level was extremely low (<0.04 ng/mL), suggesting near-complete destruction of thyroid follicles and an inability to synthesize Tg. This finding provides direct evidence of immune-mediated destructive thyroiditis rather than merely functional hypothyroidism. Following treatment with levothyroxine and methylprednisolone, Tg levels did not recover, further corroborating the sever damage to thyroid follicles.

With PD-1 inhibitor monotherapy, the incidence of hypophysitis (approximately 0.4%) is considerably lower than that of primary hypothyroidism (approximately 5%-10%) (14, 15). Therefore, the concurrent occurrence of these two conditions is relatively uncommon and likely represents two independent but coexisting immune-related adverse events. One involves T-cell-mediated immune injury to the pituitary gland (16), resulting in central hypothyroidism; the other involves immune-mediated destructive thyroiditis that directly damages the thyroid gland (17), leading to primary hypothyroidism. These are not sequential consequences of a single immune mechanism, but rather parallel attacks by the immune system on distinct endocrine organs-the pituitary and the thyroid-following PD-1 pathway inhibition.

On admission, ACTH and cortisol were markedly low; however, the 8:00 AM cortisol level on February 27 (26.94 μg/dL, near the upper normal limit) likely reflected residual exogenous glucocorticoids or a falsely low ACTH measurement. Repeat testing confirmed secondary adrenal insufficiency. The presenting hyponatremia (Na^+^ 124.6 mmol/L) with normal potassium (K^+^ 3.9 mmol/L)is consistent with this diagnosis, as the renin-angiotensin-aldosterone axis remains intact in secondary adrenal insufficiency (18). This case highlights that in elderly lung cancer patients receiving ICI, symptoms such as fatigue, lethargy, and hyponatremia should prompt a comprehensive screen for electrolyte disturbances, cardiac biomarkers, and endocrine dysfunction.

In this case, the simultaneous involvement of the ACTH and gonadal axes is consistent with diffuse anterior pituitary insufficiency caused by hypophysitis, with the growth hormone axis also being affected as part of this process. Multiple studies have shown that androgen replacement therapy can improve quality of life, mood, and libido in patients with primary or secondary adrenal insufficiency (19). In response to the patient’s low testosterone levels, oral testosterone undecanoate was initiated and gradually titrated upward; subsequent follow-up testing confirmed that testosterone levels had returned to normal.

Myxedema coma presents with depressed consciousness, hypothermia, and hypoventilation-the latter stemming from central respiratory depression, macroglossia obstruction, and respiratory muscle weakness (20, 21). The patient had somnolence, tongue-base collapse, CO_2_ retention, and pulmonary exudates on CT, indicating multifactorial type II respiratory failure, with infection, upper airway obstruction, respiratory muscle weakness, and probable severe hypothyroidism as contributing factors. Since the family declined intubation, we employed a nasopharyngeal airway, high-flow oxygen, and gradual levothyroxine titration (up to 300 μg/day), which progressively improved ventilation. This experience suggests that, for patients with respiratory failure in whom severe hypothyroidism is a probable contributing factor and who refuse invasive ventilation, such conservative measures combined with active hormone replacement may be a feasible alternative.

ICI-related myocarditis, though rare (0.7%-1%), carries a mortality of 20%-50% and frequently overlaps with myositis—particularly ominous when respiratory muscles are involved (22). Diagnosis relies on cardiac biomarkers and ECG (23, 24). In our patient, markedly elevated CK, D-dimer, CK-MB, Mb, and BNP, along with first-degree atrioventricular block, widened P waves, and myopathic EMG changes, supported the diagnosis. These findings, combined with negative work-ups for pulmonary embolism, thrombosis, renal dysfunction, tumor progression, and autoimmune antibodies, clinically diagnosed severe ICI-related myocarditis and myositis.

This case exhibited a classic irAE flare upon glucocorticoid tapering: although initial steroid therapy promptly resolved myocarditis and myositis, the patient did not complete the maintenance course, and within three weeks of discontinuat (25) multisystem injury recurred with new-onset hypophysitis, permanent thyroid destruction, and adrenal insufficiency.

Glucocorticoids are the first-line therapy for moderate-to-severe irAEs, acting through T-cell inhibition and suppression of inflammato kines (25, 26). However, PD-1 receptor occupancy can remain high for ≥57 days even after serum drug levels decline (27), indicating a prolonged immunological effect. Glucocorticoids suppress clinical symptoms without eliminating the underlying T-cell hyperactivation; rapid tapering or withdrawal therefore provokes an recurrence of ICI-related toxicity, as occurred in this case (28). Balancing immunosuppression with antitumor efficacy remains a key consideration (29). Drawing on our experience with this case, glucocorticoid therapy for MO-irAEs should adhere to the following principles (14): (i) adequate initial dosing according to toxicity grade; (ii) a treatment course of at least 4-6 weeks; (iii) gradual tapering (weekly dose reductions ≤10%-25%); (iv) close monitoring of inflammatory markers and end-organ function during tapering; and (v) long-term hormone replacement for endocrine irAEs, with extended follow-up to prevent recurrence or rebound.

Intermediate-acting glucocorticoids (e.g., prednisone or methylprednisolone) are preferred to balance efficacy and minimize HPA-axis suppression. For endocrine irAEs, which are predominantly antibody-mediated and cause secretory deficits, physiological hormone replacement is the mainstay; In patients with hypophysitis and substantial endocrine dysfunction, with or without severe headache, and who are otherwise in good general health, a course of oral glucocorticoids at 0.5–1 mg/kg/day for 6–12 months may be considered, depending on clinical severity and response (30, 31). However, this recommendation should not be confused with physiological replacement, and the dose must be individualized. In this context, the patient had shown a marked decline in myocarditis-related markers after prior glucocorticoid use, suggesting steroid sensitivity. Upon this admission, the patient was assessed as having severe myocarditis, for which high-dose glucocorticoid pulse therapy combined with intravenous immunoglobulin would theoretically be indicated. Nevertheless, because of a positive fecal occult blood test and high bleeding risk, we opted for methylprednisolone at a moderate dose (40 mg/day) rather than pulse therapy. This moderate-dose regimen was chosen to balance control of myocarditis, myositis, and systemic inflammation against the risk of further HPA-axis suppression and bleeding. This approach not only improved endocrine function but also normalized cardiac and muscle markers, validating the individualized strategy.

The management of secondary pulmonary infections during the clinical course also posed a significant challenge in this case. A study of lung cancer patients showed that the incidence of severe infections during ICI therapy can be as high as 26.90%, with bacterial infections accounting for 85% (32). Immunosuppressive therapy for irAEs markedly increases infection risk-glucocorticoids can impair neutrophil function, promote T-cell apoptosis, and compromise respiratory mucosal barrier function. In this patient, the CD4^+^T-cell count was only 240 cells/uL, indicating an immunosuppressed state and placing him at high risk for opportunistic and nosocomial infections. Consequently, he developed infections with CRAB and CRKP during the disease course.

The management of subsequent CRO pneumonia represents a translational success of precision medicine. The application of mNGS enabled rapid identification of CRAB and CRKP, guiding the rational use of eravacycline and ceftazidime-avibactam. Eravacycline is notable for its superior lung tissue penetration and 2- to 8-fold greater *in vitro* activity against CRAB compared to tigecycline (33), while ceftazidime-avibactam provided robust coverage against KPC-producing CRKP (34, 35). This targeted antimicrobial regimen, along with tailored hormone replacement, successfully reversed the multi-organ dysfunction and pulmonary infection.

Several limitations of this study should be acknowledged. First, because the patient had a metal dental prosthesis, MRI was contraindicated, and neither pituitary MRI nor cardiac MRI could be performed. Consequently, the diagnosis of ICI-related myocarditis was clinically probable rather than definitively confirmed. It was based on the temporal relationship with serplulimab, elevated cardiac biomarkers, new electrocardiographic abnormalities, and the exclusion of other possible causes; endomyocardial biopsy was also not performed. In addition, because pituitary MRI could not be performed to assess morphological changes of the pituitary gland, the diagnosis of hypophysitis was based solely on clinical and biochemical evidence, representing a clinical suspicion rather than a pathologically confirmed diagnosis. Second, serum drug levels of serplulimab were not measured, and that antigen-specific T-cell responses were not assessed. Third, as this is a single case report, the generalizability of the treatment strategy and efficacy data is limited, and further validation through multicenter, large-cohort studies is warranted.

## Conclusion

This case report describes the complete rescue process of MO-irAEs induced by serplulimab in a patient with ES-SCLC. After the patient self-discontinued glucocorticoid therapy, sever damage to multiple endocrine glands developed, followed by multifactorial type II respiratory failure with a probable contribution from severe hypothyroidism, complicated by severe pneumonia caused by carbapenem-resistant organisms. Through individualized immunosuppressive glucocorticoid therapy for ICI-related toxicity, comprehensive endocrine hormone replacement, and stepwise anti-infective therapy guided by mNGS, the patient was ultimately successfully rescued. This case highlights that clinicians using ICI such as serplulimab should maintain a high index of suspicion for severe irAEs, particularly multi-endocrine gland injury. Once suspected symptoms emerge, prompt multidisciplinary collaboration should be initiated, along with comprehensive endocrine function assessment and pathogen detection. Early recognition, standardized glucocorticoid tapering, and precise antimicrobial therapy are key to improving prognosis.

## Data Availability

The original contributions presented in the study are included in the article/**Supplementary Material**. Further inquiries can be directed to the corresponding author.
