# Supplementary material for: Immune checkpoint inhibitor-induced multi-organ immune damage complicated by severe pneumonia: case report
Source: Front Immunol. 2026 Oct 21;17:2003924. doi: 10.3389/fimmu.2026.2003924 (PMC13731381; doi:10.3389/fimmu.2026.2003924)
Supplement: Supplementary file 1 [file Table1.doc]

**Supplementary**

Table S1. Serial pituitary, adrenal, gonadal and growth hormone biomarkers over clinical course.

| **Indicator** | **COR**  **(ug/dL)** | **ACTH**  **(pg/mL)** | **GH**  **(pg/mL)** | **FSH**  **(mIU/mL)** | **PRL**  **(ng/mL)** | **LH**  **(mIU/mL)** | **T**  **(ng/mL)** | **P**  **(ng/mL)** | **E2**  **(pg/mL)** | **SHBG**  **(nmol/L)** | **IGF-1**  **(ng/mL)** | **IGFBP-1**  **(ng/mL)** |
| --- | --- | --- | --- | --- | --- | --- | --- | --- | --- | --- | --- | --- |
| **Normal range** | 7-9AM: 4.26-24.85 | 7-9AM：7.2-63.4 | 0.03-2.47 | 1.5-12.4 | 4.04-15.2 | 1.7-8.6 | 1.93-7.4 | ＜0.05-0.149 | 8.14-52.13 | 20.6-76.7 | 54.1-184 | 1839-5416 |
| **02/27/25** | 26.94 | 5.748 | 1.27 | 11.2 | 1.86 | 3.36 | 0.254 | 0.28 | 38.9 |  | 18.3 | 480 |
| **03/05/25** | 2.956 |  | 0.819 | 12.1 | 19 | 1.75 | 0.598 | 0.07 | 19.7 | 14.3 | 56.6 | 1084 |
| **03/11/25** | 2.086 | ＜1 | 0.422 | 11.2 | 16.3 | 3.24 | 0.385 | ＜0.05 | 13.2 | 11.4 | 62.1 | 1039 |
| **03/18/25** | 1.377 | 1.005 | 0.537 | 5.26 | 17.7 | 0.67 | 0.248 | ＜0.05 | 5.96 | 11 | 54.7 | 950 |
| **03/21/25** | 1.289 | ＜1 | 0.471 | 8.49 | 22.6 | 2.02 | 0.376 | ＜0.05 | 13.4 | 11 | 65 | 1195 |
| **03/24/25** | 1.385 | 1.29 | 0.513 | 7.33 | 21.1 | 0.49 | 0.546 | ＜0.05 | 27.3 | 11.8 | 68.4 | 1206 |
| **03/27/25** | 9.485 | 1.839 | 0.692 | 2.32 | 13.6 | ＜0.3 | 1.88 | ＜0.05 | 36.3 | 9.2 | 35 | 629 |
| **03/31/25** | 2.574 | 4.347 | 0.701 | 1.57 | 14 | ＜0.3 | 0.795 | ＜0.05 | 18.4 | 9.1 | 35.5 | 684 |
| **04/03/25** | 1.434 | 1.816 | 0.78 | 1.78 | 14.5 | ＜0.3 | 1.47 | ＜0.05 | 17.5 | 10 | 36.6 | 801 |
| **04/08/25** | 7.013 | 11.306 | 0.555 | 0.93 | 18.1 | ＜0.3 | 2.16 | ＜0.05 | 20.5 | 10.5 | 33.1 | 760 |
| **04/11/25** | 9.697 | 14.917 | 0.314 | 1.18 | 17.3 | ＜0.3 | 2.85 | 0.07 | 25 | 8.5 | 36.7 | 683 |

COR: Cortisol; ACTH: Adrenocor ticotropic hormore; GH: Growth Hormone; FSH: Follicle stimulating hormone; PRL: Prolactin; LH: Luteinizing hormone; T: Testosterone; P: Progesterone; E2: Estradiol; SHBG: Sex hormone binding globulin; IGF-1: Insulin-like growth factor 1; IGFBP-1: Insulin-like growth factor-binding protein-1. Reference ranges for cortisol and ACTH differ by sampling time window: morning samples collected at 7–9 a.m.

Table S2. Serial thyroid function, thyroid autoantibodies and parathyroid hormone measurements.

| **Indicator** | **T3**  **(ng/mL)** | **T4**  **(ug/dL)** | **FT3**  **(pg/mL)** | **FT4**  **(ng/dL)** | **TSH**  **(uIU/mL)** | **Tg**  **(ng/mL)** | **PTH**  **(pmol/L)** | **TGAb**  **(IU/mL)** | **TPOAb**  **(IU/mL)** | **TRAb**  **(IU/L)** | **R T3**  **(ng/mL)** | **TBG**  **(ug/mL)** | **TSAb**  **(IU/L)** |
| --- | --- | --- | --- | --- | --- | --- | --- | --- | --- | --- | --- | --- | --- |
| **Normal range** | 0.8-2.0 | 5.1-14.1 | 2.0-4.4 | 0.92-1.68 | 0.27-4.2 | 3.5-77 | 1.6-6.9 | 0-115 | 0-34 | 0-1.75 | 0.31-0.95 | 14-31 | ≤0.55 |
| **0/27/25** | ＜0.2 | ＜0.42 | ＜0.39 | ＜0.04 | 20.2 | ＜0.04 |  |  |  |  |  |  |  |
| **02/28/25** | ＜0.2 | ＜0.42 | ＜0.39 | ＜0.04 | 46.8 | ＜0.04 | 3.05 | 31.6 | 239 | 0.94 | ＜0.05 | 12.8 | 4.49 |
| **03/05/25** | ＜0.2 | 1 | ＜0.39 | 0.12 | 99 | ＜0.04 |  |  |  |  |  |  |  |
| **03/11/25** | ＜0.2 | 1.4 | ＜0.39 | 0.21 | 51.3 | ＜0.04 |  |  |  |  |  |  |  |
| **03/18/25** | 0.25 | 2.85 | 0.86 | 0.66 | 43.9 | ＜0.04 |  |  |  |  |  |  |  |
| **03/21/25** | 0.26 | 4.4 | 1.14 | 1.08 | 26.9 | ＜0.04 |  |  |  |  |  |  |  |
| **03/24/25** | 0.27 | 6.89 | 1.13 | 1.57 | 15.3 | ＜0.04 |  |  |  |  |  |  |  |
| **03/27/25** | 0.22 | 6.89 | 0.91 | 1.87 | 1.1 | ＜0.04 |  |  |  |  |  |  |  |
| **03/31/25** | 0.29 | 7.46 | 1.05 | 2.36 | 0.24 | ＜0.04 |  |  |  |  |  |  |  |
| **04/03/25** | 0.3 | 5.24 | 1.31 | 1.8 | 0.205 | ＜0.04 |  |  |  |  |  |  |  |
| **04/08/25** | 0.2 | 2.85 | 0.68 | 0.92 | 2.6 | ＜0.04 | 1.58 |  |  |  |  |  |  |
| **04/11/25** | 0.24 | 3.71 | 0.81 | 1.19 | 2.99 | ＜0.04 |  |  |  |  |  |  |  |
| **04/14/25** | ＜0.2 | 5.02 | 0.86 | 1.82 | 1.38 | ＜0.04 |  |  |  |  |  |  |  |

T3: Triiodothyronine; T4: Thyroxine; FT3: Free Triiodothyronine; FT4: Free Thyroxine; TSH: Thyrotropin; Tg: Thyroglobulin; PTH: Parathyroid hormone; TGAb: Thyroglobulin antibody; TPOAb: Thyroid peroxidase antibody; TRAb: Thyrotropin receptor antibody; R T3: Reverse triiodothyronine; TBG: Thyroxine-binding globulin; TSAb: Thyroid-stimulating antibody;

Table S3. Serial liver, renal, electrolyte, inflammatory and complete blood count parameters.

| **Indicator** | **ALT**  **(U/L)** | **AST**  **(U/L)** | **r-GGT**  **(U/L)** | **ALP**  **(U/L)** | **LDH**  **(U/L)** | **Scr**  **(umol/L)** | **K^+^**  **(mmol/L)** | **Na^+^**  **(mmol/L)** | **Cl^-^**  **(mmol/L)** | **WBC**  **(×10^9/L)** | **NEUT%**  **(%)** | **CRP**  **(mg/L)** | **SAA**  **(mg/L)** | **PCT**  **(ng/mL)** | **IL-6**  **(pg/mL)** |
| --- | --- | --- | --- | --- | --- | --- | --- | --- | --- | --- | --- | --- | --- | --- | --- |
| **Normal range** | ＜50 | 17-59 | 15-73 | 38-126 | 120-246 | 58-110 | 3.5-5.1 | 137-145 | 98-107 | 3.5-9.5 | 40-75 | 0-10 | ＜10 | ＜0.046 | 0-7 |
| **02/26/25** | 50 | 49.5 | 36.9 | 73.5 | 355 | 88.3 | 3.87 | 124.58 | 93.85 | 5.63 | 84.5 | 78.78 | 145.91 | 0.116 | 32.86 |
| **02/27/25** | 42 | 38.8 | 32.8 | 71.3 | 297.5 | 114.1 | 4.24 | 127.51 | 95.48 | 5.22 | 83.3 | 68.33 | 84.33 | 0.324 |  |
| **02/28/25** | 33 | 31.2 | 27.8 | 61.4 | 271.1 | 109.6 | 4.25 | 128.87 | 97.74 | 5.23 | 84.2 | 39.79 | 37.36 | 0.3 | 5.51 |
| **03/03/25** | 32 | 31.3 | 24.6 | 72.6 | 237.9 | 100.4 | 3.47 | 131.03 | 87.71 | 6.27 | 80.7 | 15.53 | 29.42 | 0.143 | 10.48 |
| **03/07/25** | 43 | 37 | 30.9 | 74.8 | 282.7 | 95.7 | 4.51 | 136.95 | 93.34 | 5.93 | 78.8 | 8.61 | 18.39 | 0.112 | 15.61 |
| **03/10/25** | 36 | 25.9 | 26.7 | 65.9 | 229.4 | 95.6 | 4.34 | 138.73 | 94.44 | 5.48 | 73.9 | 5.77 | 19.49 | 0.231 | 9.54 |
| **03/13/25** | 40 | 33.7 | 26.6 | 69.8 | 229 | 82.7 | 4.45 | 136.16 | 94.34 | 3.96 | 79.5 | 5.3 | 24.72 | 0.106 | 2.86 |
| **03/17/25** | 41 | 31.1 | 26.3 | 67.3 | 230.5 | 78.8 | 4.4 | 133.51 | 95.91 | 4.4 | 69.7 | 3.19 | ＜8 | 0.076 | ＜1.5 |
| **03/20/25** | 40 | 35.8 | 32.2 | 75.1 | 241.3 | 84.6 | 4.21 | 137.59 | 98.07 | 4.19 | 71.6 | 4.13 | ＜8 | 0.091 | 2.73 |
| **03/24/25** | 43 | 33.3 | 36.7 | 71.2 | 281.8 | 79 | 4.21 | 139.19 | 100.96 | 5.52 | 70.7 | 2.9 | ＜8 | 0.068 | 4.85 |
| **03/25/25** | 35 | 26 | 34.5 | 69.7 | 231.5 | 101.5 | 4.56 | 140.33 | 103.82 | 7.29 | 75.9 | 7.38 | 60.76 | 1.07 | 617.4 |
| **03/26/25** | 38 | 25 | 31.8 | 64.4 | 227 | 89.8 | 4.21 | 142.76 | 106.73 | 7 | 70.8 | 80.19 | 195.75 | 1.73 | 63.02 |
| **03/27/25** | 37 | 21.9 | 27.9 | 70.2 | 207.1 | 71.9 | 4.01 | 143.24 | 104.81 | 5.62 | 76 | 77.16 | ＞288 | 0.975 | 16.52 |
| **03/31/25** | 34 | 18.3 | 23.3 | 73.8 | 174.6 | 63.5 | 4.22 | 141.71 | 103.67 | 4.21 | 72.9 | 8.88 | 17.33 | 0.081 | 6.06 |
| **04/02/25** | 27.9 | 17.2 | 24 | 61 | 211 | 54.4 | 4.31 | 141.25 | 101.61 | 3.91 | 69.8 | 4.44 | 9.3 | 0.074 | 9.88 |
| **04/07/25** | 32 | 19.6 | 28.1 | 73.3 | 212.2 | 55.9 | 4.57 | 144.59 | 105.68 | 6.55 | 76.2 | 3.32 | 10.71 | 0.055 | 3.9 |

ALT: Alanine aminotransferase; AST: Aspartate aminotransferase; γ-GGT: Gamma-glutamyl transferase; ALP: Alkaline phosphatase; LDH: Lactate dehydrogenase; Scr: Serum creatinine; K⁺: potassium; Na⁺: sodium; Cl⁻: chloride; WBC: White blood cell count; NEUT%: Neutrophil percentage; CRP: C-reactive protein; SAA: Serum amyloid A; PCT: Procalcitonin; IL-6, Interleukin-6.

Table S4. Serial cardiac injury and heart failure biomarker measurements.

| **Indicator** | **CK (U/L)** | **hs-TnT(ng/mL)** | **CK-MB (ng/mL)** | **Mb (ng/mL)** | **NT-proBNP (ng/L)** |
| --- | --- | --- | --- | --- | --- |
| **Normal range** | 50-310 | ＜0.014 | ＜4.87 | 28-72 | ＜125 |
| **02/26/25** | 438 | 0.292 | 19.22 | 133.6 | 239.4 |
| **02/27/25** | 192 | 0.181 | 7.28 | 64.09 | 144.6 |
| **03/05/25** | 98 | 0.137 | 3.73 | 61.65 | 63.76 |
| **03/07/25** | 104 | 0.099 | 5.93 | 62.14 | 116.7 |
| **03/11/25** | 40 | 0.086 | 1.96 | 38.73 | 246.6 |
| **03/13/25** |  | 0.06 | 1.67 | 47.53 | 106.2 |
| **03/17/25** |  | 0.048 | 2.15 | 38.15 | 119.7 |
| **03/20/25** |  | 0.038 | 2.42 | 38.71 | 145.3 |
| **03/24/25** |  | 0.043 | 2.7 | 41.35 | 174.6 |
| **03/25/25** |  | 0.059 | 2.07 | 51.33 | 801.7 |
| **03/26/25** |  | 0.052 | 0.651 | 43.79 | 348.9 |
| **03/27/25** |  | 0.037 | 0.7 | 44.66 | 180.8 |
| **03/31/25** |  | 0.027 | 0.723 | 47.02 | 415.6 |
| **04/02/25** |  | 0.026 | 1.02 | 49.43 | 369.7 |
| **04/07/25** | 26 | 0.022 | 0.819 | 69.58 | 200 |
| **04/09/25** |  | 0.027 | 0.802 | 95.65 | 194.5 |

CK: Creatine Kinase; hs-TnT: High-sensitivity troponin T; CK-MB: Creatine Kinase-MB; Mb: Myoglobin; NT-proBNP: N-terminal pro-B-type natriuretic peptide
